# System steganalysis with automatic fingerprint extraction

**DOI:** 10.1371/journal.pone.0195737

**Published:** 2018-04-25

**Authors:** Alejandro Cervantes, Tom Sloan, Julio Hernandez-Castro, Pedro Isasi

**Affiliations:** 1 University Carlos III of Madrid, Leganés, Madrid, Spain; 2 University of Kent, Canterbury, United Kingdom; George Mason University, UNITED STATES

## Abstract

This paper tries to tackle the modern challenge of practical steganalysis over large data by presenting a novel approach whose aim is to perform with perfect accuracy and in a completely automatic manner. The objective is to detect changes introduced by the steganographic process in those data objects, including signatures related to the tools being used. Our approach achieves this by first extracting reliable regularities by analyzing pairs of modified and unmodified data objects; then, combines these findings by creating general patterns present on data used for training. Finally, we construct a Naive Bayes model that is used to perform classification, and operates on attributes extracted using the aforementioned patterns. This technique has been be applied for different steganographic tools that operate in media files of several types. We are able to replicate or improve on a number or previously published results, but more importantly, we in addition present new steganalytic findings over a number of popular tools that had no previous known attacks.

## Introduction

Steganalysis is the counterpart to steganography. Its goal is to prove that two parties are communicating by using steganography, where embedding algorithms can hide information by employing a wide variety of techniques. To be successful, a steganalytic algorithm must be capable of distinguishing between stego-objects and cover-objects with a probability higher than that of a random guess [1]. In a practical setting, where millions of files need to be analyzed, this implies that much higher accuracy rates are really needed. This is especially true when taking into account real investigative constraints and their impact on time and other limited resources. This will often mean that a steganalytic attack needs to be developed and carried out both quickly and effectively to be of any use in a real setting to forensic practitioners and law enforcement.

One can argue that the purpose of steganalysis is to find consistent signatures, if the concept of a ‘signature’ is sufficiently ample. A signature defined in this fashion is any change that is consistently introduced on the target (stego-object) due to the steganographic process. The presence or absence of the signature can then be used to reliably decide if a given object has some hidden content.

To achieve this, two major approaches exist within steganalysis: statistical and system attacks [2]. Statistical attacks analyse the signal component of the object, ignoring other information that can be present in the stego-file. For instance, one particular approach may study the relationships between the DCT coefficients of a JPEG file [3], while for video files a steganalytic scheme might focus on a specific component of the motion vectors [4]. Each format requires an independent approach. Often, statistical attacks use high-order statistics for training classifiers to detect statistical differences between stego-objects and cover-objects.

In a practical setting, statistical steganalysis may have limitations that make their attacks impractical during a standard investigation. Statistical attacks are often highly reliable against one particular scheme, providing a high degree of accuracy. This is often restricted to a single steganography scheme and such attacks can be time-consuming to develop and test. Statistical attacks often assume a pre-existing knowledge of the steganographic scheme, which may not always be applicable in a real-world investigation where only the stego-object is provided. Furthermore, general strategies exists that, by introducing subtle modifications to a given embedding scheme, can provide greater resistance against statistical attacks [5].

System steganalysis, instead, focuses on implementation vulnerabilities to attack a steganographic scheme [6]. System-based approaches usually analyze the whole stego-object as opposed to focusing only on the embedding algorithm or the proper media contents. In steganography, an *ideal* scheme would only modify the intended components of its target object. However, in practice, stego-systems can often introduce unintentional modifications in other components of the object. System attacks are able to detect these unintentional modifications. They can sometimes lead to steganalytic attacks that offer greater accuracy and efficiency. Thus, system attacks can at times be used to circumvent the practical limitations of statistical steganalysis. Moreover, they do not rely so heavily on pre-existing knowledge of the steganographic scheme. System and statistical attacks are very complementary of one-another. In scenarios where one does not work, the other can frequently offer a practical alternative.

In this paper, we develop a framework for automatic system steganalysis that is based on the construction of a classifier able to detect implementation vulnerabilities introduced by stego-systems. This classifier operates on attributes that are extracted from the training data using pattern-matching processes. The choice of classifier is a well-known technique in machine learning (Naive Bayes classifier), but it provides additional flexibility over a simple signature-matching system.

The framework has been applied successfully to a wide variety of schemes and across different formats because it has been designed to be as general as possible. Hence, it makes very few assumptions on the underlying nature of the stenography tool or cover media being used.

The resulting framework has several improvements on previous work in this field. First, it decouples attribute extraction from classification. Also, we propose a generalization phase that is able to improve results, provided that training media objects are representative of real-world media objects. Finally, the trained classifier can operate even when some of the obtained fingerprints for a given steganographic tool are not present in observed media, as the constructed model may combine several different findings for a given tool.

The rest of the paper is structured as follows. Section 2 provides a discussion of related work in the field. Section 3 gives a comprehensive insight into how we build the classifier and carry out feature extraction using the framework that we have built, and describes how it operates. Section 4 applies our framework to a series of datasets created with several steganography tools. In this section, we discuss and evaluate the performance of our approach and provide a general discussion of its limitations. We conclude the paper in Section 5, where we present some final thoughts and insights and discuss future avenues of work.

## Related work

### System steganalysis

System attacks rely on unintentional implementation vulnerabilities that are often relatively independent of the steganographic scheme. This has been briefly discussed before. Our previous research [7, 8] used system-type attacks to examine and evaluate the current state of steganography tools, with a focus on video-based applications. We found that many of the most accessible and popular tools share similar embedding schemes and that they were highly susceptible to system steganalysis. We have, in addition, carried out research on the popular steganography & watermarking tool, OpenPuff. Our findings led us to believe that its video embedding scheme would follow a similar scheme, which is unfortunately common in other stego tools operating over video formats. This is one particular theory we examine throughout this paper.

Chen et al. have discussed the concept of file structure steganography and its application to images, and have shown how implementation vulnerabilities can be used to reliably find signatures [9]. They noted that one particular advantage of this type of approach is that it is not affected by techniques typically employed to increase resistance against statistical steganalysis. Cantrell examined file-structure based steganography, with a focus on digital documents [10]. This work further emphasized the need for steganalytic systems that exploit the very common vulnerabilities arising from a poor handling of metadata during the steganographic process.

System attacks have already been successful in the field of steganalysis. In [11] the authors proposed a type of attack that is aimed to detect the use of steganographic software independently of the actual media being used. The process simply compares the average values of bits at fixed positions of both an unmodified object and the corresponding stego-object when subject to the steganographic tool. Results showed that many tools were introducing anomalies that could be easily used to detect stego-objects. However it must be noted that if the anomaly is not present at the exact same position in all files, this method can’t detect it. Also, objects are only labeled as stego-objects if all the regularities are present, due to the problem of false positives.

Another work [12] extends this idea to the search of binary or hexadecimal patterns by proposing a recognizing automaton that can also find regularities that are not restricted to specific positions in the analyzed objects. This method was used for steganography on image files, and different policies are defined for the header, content, and tail parts of those file formats.

Our implementation of sequence extraction is a further step in this type of approach. However, it is more powerful in several senses. First, we are using a formal definition of a pattern by using a specific grammar. This makes clear the type of regularities that our system is able to detect. A more complex grammar might be used with increased detection capabilities. Similarly to the most recent work [12], the process of pattern extraction and verification is not restricted to specific positions in the file. However in our case we don’t make assumptions on the part of the file that is being explored. This allows this approach to work with a broad range of different file formats. Third, in our approach we separate pattern extraction and classification phases, so both components can be substituted if required. Finally, we generate a probabilistic classifier that is more complex than simple signature detection, as it is able to perform its task using a combination of patterns, that are not necessarily present in all the analyzed objects. Even though our classifier does not work in that way, in the discussion of the results we also report the cases where classical signatures were found. We test this method on several steganographic tools not covered in previous works. and we have also been able to apply it regardless of media format.

### Overview of Bayesian classification

Bayesian Probabilistic Classification is a broad but well-established research area with algorithms currently deployed in many different real-world scenarios over different domains. A very generic description for all such systems would be that they rely on the construction of a Bayesian Network [13] that takes as evidence a feature set extracted from the data, and can be queried to make a prediction for the class to which data belongs. Prior knowledge of the problem can be used to determine the structure of the network, and known data is employed to learn the network parameters, the structure and/or the Conditional Probability Tables (CPTs) required to execute queries. In addition, some approaches employ Markov Chains to be able to reflect the temporal dimension. A good introduction Bayesian Probabilistic Classification can be found in [14].

Similar strategies have recently been used in a computer security context. For instance, in [15] the authors construct a Network Intrusion Detection System (NDIS) that uses byte patterns as attack signatures for analyzing network traffic and generating alerts. This Bayesian Network can even be dynamic (providing stateful detection) [16]. However, learning the parameters for the Bayesian Network can be a very complex process. Also, there are circumstances in which we do not have *a priori* knowledge of the best structure for the network. For these reasons, simpler approaches are sometimes used, and can also lead to good results. For example, in [17–19] the authors preferred to employ a Naive Bayes Classifier [20], which is equivalent to a simple Bayesian Network with no inter-feature dependencies.

For our work, we can formulate the steganalysis problem as a binary classification problem, where we define that cover-objects belong to *Class*_0_ (or negatives) and stego-objects belong to *Class*_1_ (or positives). Each object (*X*_*i*_) is represented by a set of up to *N* attributes (*X*_*i*_ = {*A*_*i*1_, *A*_*i*2_, ⋯, *A*_*iN*_}).

The principle of Bayesian classification is to estimate the posterior class probability of an observation *X*_*i*_ using the empirical probabilities obtained from a previous sample of the domain. For any class *k*, *Pr*(*Class*_*k*_|*X*_*i*_) can be estimated from *Pr*(*X*_*i*_|*Class*_*k*_) using the Bayes Theorem *Pr*(*Class*_*k*_|*X*_*i*_) = *αPr*(*Class*_*k*_)*Pr*(*X*_*i*_|*Class*_*k*_), where *α* = 1/*P*(*X*_*i*_) is independent of the class.

In the general case, obtaining *Pr*(*X*_*i*_|*Class*_*k*_) is not a trivial task because the distributions of values for features may have multiple dependencies and correlations among them. However, our previous experience in this domain suggests that this needs not be the case for the problem we are trying to solve. Also, a simpler model can be more accommodating to analyze a broad class of steganographic schemes, as features are not the same for all schemes. For these reasons this work uses the simplest approach to Bayesian classification, the well known Naive (or Simple) Bayes classifier [20].

This approach is based on a strong assumption, which is the conditional independence of the distribution of values for each attribute (Eq 1). This kind of model is simple enough to train, and has proved to be reliable even in scenarios where it is well known that there is at least a partial violation of its theoretical assumptions [21].

$$\begin{array}{c} Pr\left(X_{i}|{Class}_{k}\right)=\prod_{j\in \{1..N\}}Pr\left(A_{ij}|{Class}_{k}\right) \end{array},$$ (1)

where *Pr*(*A*_*ij*_|*Class*_*k*_) is an abbreviated notation for the probability of obtaining the particular value *A*_*ij*_ as the *j*–th attribute for any object of class Class_*k*_.

The most probable class is assigned to an unknown observation (Eq 2). If classes are balanced then *αPr*(*Class*_*k*_) is also constant, and only in this case the class can be selected by using the one producing a maximum product of likelihoods. This is called the Maximum Likelihood Rule.

In our particular domain, the values for each feature are obtained by finding the matches of a set of patterns in the file extracted from training data (shown later in this paper). The resulting *N* attributes are used then to predict the class of the file using Eq (2).

$$\begin{array}{c} Class\left(X_{i}\right)=\arg\max_{k\in \{0,1\}}\prod_{p\in Model}Pr\left({Class}_{k}\right)\times {Pr}_{p}\left(A_{ip}|{Class}_{k}\right) \end{array},$$ (2)

where each factor *Pr*_*p*_(*A*_*ip*_|*Class*_*k*_) is the probability of obtaining the value *A*_*ip*_ in files of class *Class*_*k*_ when we apply the pattern *p* to a file *X*_*i*_.

Let us say that the possible values obtained when matching pattern *p* on the available objects are *v*_*pt*_ ∈ {*v*_*p*1_, *v*_*p*2_, ⋯}. Then, the task of model construction becomes equivalent to obtaining the probability distributions *Pr*_*p*_(*v*_*pt*_|*Class*_*k*_), for each specific value *v*_*pt*_. This can be done by replacing the actual distribution by the empirically estimated $Pr_{p}^{*}\left(v_{pt}|{Class}_{k}\right)$, calculated as a frequency. First, each pattern is matched against both a set *Set*_0_ of cover-objects and a set *Set*_1_ of stego-objects. Then, for each value *v*_*pt*_ thus obtained, we calculate its frequency using Eq (3).

$$\begin{array}{c} Pr_{p}^{*}\left(v_{pt}|{Class}_{k}\right)=\frac{Count\left(v_{pt}\right)\;\forall objects\in {Set}_{k}}{|{Set}_{k}|} \end{array}$$ (3)

These constitute the empirical Conditional Probability Tables (CPTs) of the constructed Naive Bayes Model.

The previous concepts are used in Steps 3 and 4 of our procedure, Model Generation and Model Refining.

## Description of the framework

Previous work in system steganalysis show that, quite often, the steganography process introduces predictable changes in stego-objects. In this work we search for these modifications using a method that is independent of the exact tool or media format. The results obtained are related to the particular tool used, though sometimes several tools can be affected by the same vulnerability due to the use of common libraries. These changes extend beyond the unavoidable impact upon the signal itself, which is the subject of classical statistical steganalysis. These implementation issues are generally unintentional modifications that can be, however, very useful to distinguish between cover objects and stego-objects.

Implementation issues of this type can provide an analyst with a number of options for developing an effective steganalysis framework. Signatures which can be seen as consistent imprints from the stego-system are a classic candidate for these attacks. Signatures are mostly unique to the stego-system and appear solely in the stego-object. They often consist of strings of enough length and entropy so that perfect accuracy (in terms of zero false positives and negatives) can be achieved [9], although of course this is not always the case.

### Concepts and definitions regarding attribute extraction

In this section we use a specific example to describe some of the terms used lated when we describe the procedure itself and all its steps.

Our objective is then the construction of a classifier able to separate cover objects from stego-objects. As in any machine learning technique, objects have to be described in terms of features, also called attributes. For instance, the existence of a given signature in an object would map to an attribute that has two possible values, *True* (if the signature is present) or *False* (otherwise). A supervised classification system is able to learn the correlation between the existence of those features and each of the object classes, if provided with adequate training data. This training data would incorporate the class of each of the objects (labeled data). The information the classifier learns can be called a “model”. The final objective is twofold: to build a model that represents the information available for training, but also that successfully predicts the correct class for any previously unseen object, such as those found in a real-world scenario. This is usually called “generalization”.

Without any assumption on where potential signatures will be located in the object, attribute generation has to be modeled as a search for modifications produced by the steganographic process. This search can be performed by comparing a cover-object with its corresponding stego-object. Then, the system has to determine if the change is consistent throughout a significant part of the available labeled data. A classifier can only use these “consistent features”, because they are the ones that represent information not particular to single pairs of cover and stego-objects. For the same reason, those attributes are likely to be the most useful to construct a model able to generalize, that is, a model able to successfully distinguish between stego and cover objects in the most generalizable way.

In this work we use a simple grammar, shown in Table 1, to identify potential attributes of the classification model. With it, three different pattern types can be generated:

- Fixed patterns. Those are strings that can be found in cover objects but missing in stego objects or, more frequently, the other way around. They generally correspond to some piece of information injected or deleted by the stego process. Those patterns become attributes, labeled by the value of the pattern itself, 0012-6e4d for instance, and take binary values, True or False.
- Variable patterns. They are strings in which a particular sub-string is constantly changed by the same value or values. For instance, pattern 0012????0000????6e4d6574 describes one where the variable part ???? is systematically changed for the same value or values, but the remaining 8 bytes stay constant (0012, 0000, 6e4d6574). Variable patterns become attributes labeled with the constant part. For instance, if 0012abcd000012346e4d6574 and 001212340000abcd6e4d6574 are both found, a new attribute called 0012????0000????6e4d6574 is included, where the only values permitted are: abcd-1234, 1234-abcd and Void.
- Extended patterns. Those patterns correspond with situations where differences between the cover and stego objects include a fixed part, a variable part that changes always to the same value or values, and a variable part that takes arbitrary values. For instance, the pattern 0012????****????6e4d6574 is similar to the previous example, but without taking into account the value of the third byte.

**Table 1:** Grammar used for the patterns, in EBNF notation.

<pattern> ⩴ <cnst> [ <var> | { <mconst> } ] [ <cnst> ]
<mcnst> ⩴ <var> <cnst> <var>
<var> ⩴ <skip> [ <val> ] | <val> [ <skip> ]
<cnst> ⩴ <hex> { <hex> }
<skip> ⩴ “*” { “*” }
<val> ⩴ “?” { “?” }
<hex> ⩴ [af_09]

The search for patterns is performed by comparison of each pair of cover and stego objects, using the aforementioned grammar as reference for identifying and generating attributes.

At the end of this process of attribute discovery, the entire object dataset is replaced by an attribute-value table, where each row corresponds to an object, stego or cover, each column to an attribute, and the cells are filled with the values of each attribute for each object.

In Table 2 a learning set with 30 objects (15 stego and 15 cover) and 3 attributes is shown. For instance, column 21 corresponds to an object where the string “6e4d6574” is not present, string “00123b2a00009218” is, and “00122dfb****e05d” is also present, no matter the value assigned to the asterisks. This table constitutes the training set for the naive-bayes algorithm, which is used in the next step to distinguish between cover and stego objects. In the next section, we introduce a more detailed description about how to built the training set.

**Table 2:** Example of a learning table.

Attributes	6e4d-6574	0012-???? 0000-????	0012-???? ****-????
Objects		6e4d-6574	6e4d-6574
VideoS 1	True	Void	Void
VideoC 1	False	Void	Void
VideoS 2	True	Void	Void
VideoC 2	False	Void	Void
VideoS 3	True	Void	Void
VideoC 3	False	Void	Void
VideoS 4	True	Void	Void
VideoC 4	False	Void	Void
VideoS 5	False	Void	Void
VideoC 5	False	Void	Void
VideoS 6	False	3b2a-9218	Void
VideoC 6	False	Void	Void
VideoS 7	False	3b2a-9218	Void
VideoC 7	False	Void	Void
VideoS 8	False	3b2a-9218	Void
VideoC 8	False	Void	Void
VideoS 9	False	2dfb-e05d	Void
VideoC 9	False	Void	Void
VideoS 10	False	2dfb-e05d	Void
VideoC 10	False	Void	Void
VideoS 11	False	3b2a-9218	2dfb-e05d
VideoC 11	False	Void	Void
VideoS 12	False	3b2a-9218	3b2a-9218
VideoC 12	False	Void	Void
VideoS 13	False	3b2a-9218	2dfb-e05d
VideoC 13	False	Void	Void
VideoS 14	False	2dfb-e05d	3b2a-9218
VideoC 14	False	Void	Void
VideoS 15	False	2dfb-e05d	3b2a-9218
VideoC 15	False	Void	Void

### Model construction from extracted features

**Fig 1:**
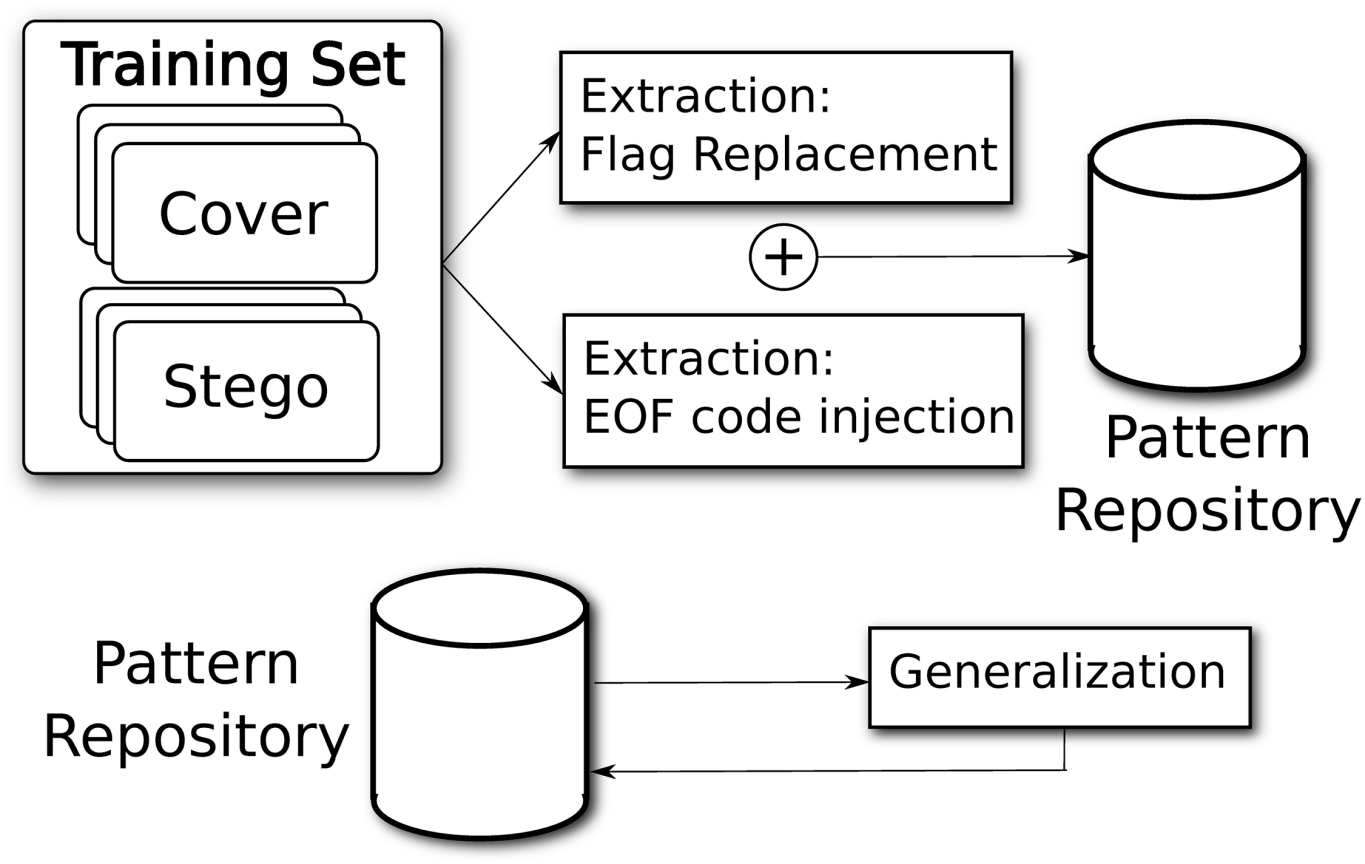
Block diagram of the process, Steps 1 and 2. Step 1, Pattern Extraction, requires pairs of files, and stores all the results in a common database; Step 2, Pattern Generalization, is executed after the pattern repository is created, and generalized patterns are added to this repository.

**Fig 2:**
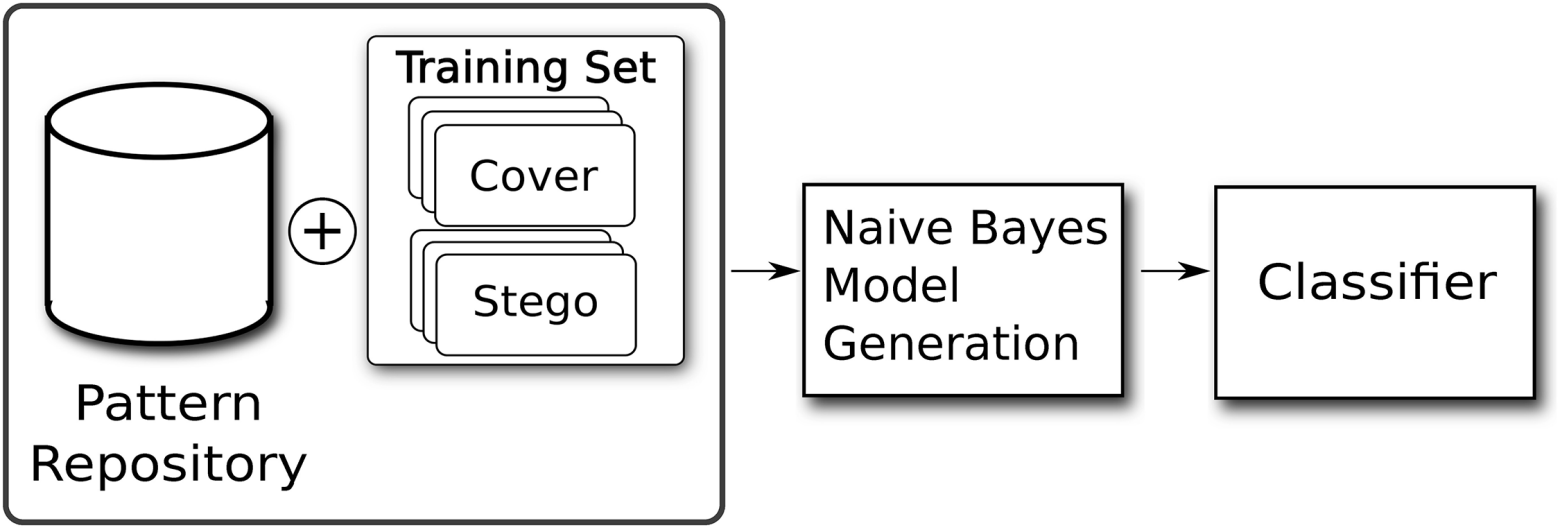
Block diagram of the process, Step 3. Model generation takes the stored patterns and builds a Naive Bayes Classifier based on the frequencies of values in the training set.

The overall process we propose in this work proceeds in five steps:

1. Extract patterns from a set of paired objects (cover and stego), each one labeled with the class it belongs to (as depicted in Fig 1).
2. Generalize patterns extracted in the previous step, and add the resulting extended patterns to the repository.
3. Build a raw model from all patterns by searching on the whole Training set (Fig 2).
4. Refine model using predefined rules.
5. Validate model to obtain the success rate on objects whose class is unknown

Note that training requires having paired objects, that is, both a cover-object and the corresponding stego-object, where a steganographic scheme has embedded a message of unknown length. This set of objects is what we are referring to as Train set.

In the experimental procedure we also use a Test Set, choosing randomly a fraction from the Train Set. The use of a separate test set is a common practice for parameter tuning and to stop training [22]. Our experiments were evaluated using k-fold crossvalidation on the third group of objects, the Validation set, so the split among Train, Test, and Validation sets is performed 10 times.

In the following sections we detail each of the aforementioned steps in the process.

### Pattern extraction and pattern generalization

#### Step 1: Pattern extraction

This phase processes files from the training set to extract patterns that can be used to construct a model. These selective patterns have to be searched using a predefined criteria so they represent the modifications that a stego-system imprints onto the files that represent both cover-objects (called cover-files) and stego-objects (called stego-files) through its embedding scheme.

In our experiments we initially searched for patterns of two types, based on previous knowledge of two typical steganography techniques, namely Flag Replacement and End-of-File code injection.

- Patterns for Flag Replacement are extracted comparing a cover-file with its corresponding stego-file. The search finds consistent sequences of bytes located in parallel between both files.
  In Table 3 we describe the extraction process of one of these patterns. In this example, we explored an object pair and detected that, at a given position we could find 11 bytes that had the same value in both files, then we had 3 bytes that were different in both files, followed by 4 bytes that had again the same value in both.
- Patterns for EOF Injection are sequences of bytes that are found anywhere (but frequently at the end) in a stego-file, but not in the corresponding cover-file. We return in this case only constant patterns, that is, patterns with no variable part.
  An example of these patterns can be found in Table 4. The final portion of the cover-file is repeated in the stego-file, but there are some additional contents present in the stego-file. We select a section of these contents (2a50523131306d622020 in the example) and store it if that fragment is not found at any position of the cover-file.

**Table 3:** Example pattern for flag replacement.

File [Pos.]	Hexadecimal dump in file order
Cover [0xa]	0000001200017b0000000000000002000a6f
Stego [0xa]	0000001200017b0000000004101002000a6f
Pattern	0000001200017b000000????????02000a6f

**Table 4:** Example pattern for EOF code injection.

File	Hexadecimal dump in file order	
Cover:	777bbbf0	EOF
Stego:	777bbbf0	2a50523131306d622020… EOF
Pattern:	2a50523131306d622020	

#### Step 2: Pattern generalization

The result of the pattern extraction process is a pattern repository. On this repository, we run a generalization process that introduces an additional number of extended patterns.

We introduced this mechanism to account to the fact that some object pairs had specific parts that were being introduced in the patterns, when considered those pairs individually, but were never repeated in other data. For instance, the file size or file name might be present in that section of the files, and would be consistent in cover and stego-objects, but would never (or rarely) be repeated in any other object. So those patters were useful, but had to be generalized to match more than one pair of cover and stego-objects.

This process is performed as follows:

1. Group the patterns in clusters, according to similarity
2. For each cluster formed that contains at least two patterns, create a generalized pattern by replacing each position that is not identical in all patterns with the (*) sign

As a clustering method, we use a hierarchical agglomerative clustering technique (HAC) with single linkage [23], employing the Hamming distance as a measure of similarity between patterns. When comparing two patterns, their Hamming distance was computed as the ratio of symbols that are different for each position in the pattern. That is, two patterns where all positions have the same symbols have distance 0.0; if a third of the symbols are different, then distance is approximately 0.33 and so on. The patterns that were closer in terms of Hamming distance were clustered together, as long as their distance was not above a certain threshold *Th*. The distance function assigned distance 1.0 to patterns of different length, thus ensuring that the cluster mechanism never placed those patterns in the same group.

As seen in Table 5, new patterns are obtained where some of the bytes are to be ignored. This step is relevant if the original patterns extracted when comparing a cover-object and its corresponding stego-object are specific only to that pair. Extended patterns are guaranteed to match at least two pairs of objects, and have much improved chances of matching new objects not included in the model generation phase.

**Table 5:** Example of the pattern generalization process. The repository contains three variable patterns, and we generate a single extended pattern.

Patterns	Hexadecimal dump in file order
1	0000001200017b000000????????02000a6f
2	0000001200027b000000????????02000a6f
3	000000120ffefb000000a5??????02000a6f
Extended	000000120****b000000**??????02000a6f

#### Step 3: Model generation

Model generation consists in the application of every pattern to all object pairs in the training set, and the computation of two distributions of values for each pattern, one for the cover-object and one for the stego-object. This is done by calculating the frequencies for the values obtained for each pattern, as described in a previous section.

An example of model generation is presented in Table 6. The first section in Table 6 corresponds to the pattern in Table 3. It matched 90% of the cover-objects and also 90% of the stego-objects.

**Table 6:** Example Model constructed from the example patterns.

Pattern 1: 0000001200017b000000????????02000a6f			
	Matches	Value	Frequency
Cover (*Class*_0_)	90%	0000 0000	0.90
		False	0.10
Stego (*Class*_1_)	90%	0000 0000	0.18
		0004 1010	0.01
		… others..	⋯
		False	0.10
Pattern 2: 2a50523131306d622020			
	Matches	Value	Frequency
Cover (*Class*_0_)	0%	False	1.0
		True	0.0
Stego (*Class*_1_)	75%	False	0.25
		True	0.75

We include the value “False” for the cases where the pattern did not match. For the cases the pattern matched, we obtain the frequencies of the values located among the training set. The result in this example was:

- For cover-objects the value was 0000 0000.
- For stego-objects, the values were 0000 0000 in 18% of cases, and different from 0000 0000 (possibly random values) in 82% of cases. This may mean that a generally unused “flag” position was frequently (but not always) modified by the embedding scheme.

The second pattern in Table 6, corresponds to the example pattern shown in Table 4. As the pattern has only a constant part, we only calculate how frequently it matches files of both classes. For our example, we assume it was never identified within cover-objects, and it was found in 75% of the stego-objects. We conventionally assign the value “False” when a pattern does not match, and “True” when it does.

These frequencies are then stored as CPTs for the model, and used for classification using the Maximum Likelihood rule (Eq 2). In the Appendix S1 Appendix we provide details of how this would be applied to a specific case using the current example.

#### Step 4: Model refining

After model construction, but before classification, we can apply some filtering to the raw model to eliminate patterns that are certainly not useful for classification. This pruning process consists in applying the following rules:

- Eliminate patterns that only match files in one file pair (the file pair from which they were obtained)
- Eliminate patterns whose distribution of values is the same for both cases (cover and stego).
- Eliminate patterns whose distribution of values is thought to be approximately random for both cases (cover and stego).

Also the following two policies were used to modify the CPTs in the model, to address some practical issues in this process of model construction:

- The empirical distribution may not be complete, in the sense that some of the values for a feature may not be found in any of the cases used for training. This is usually called the problem of zero-observation (that is, a possible value is never observed when training). In this case we adopted the simplest strategy used in literature, that is, assigning a fixed small value to the factor corresponding to unknown values. This ensures that the joint probability product does not become 0 due to unknown values [14].
- The CPT associated to a feature was transformed when the CPT for a feature had a single value *V*_0_ with frequency 1.0 in one case (either cover or stego). In these cases, the CPT was considered binary, that is, with only two cases: *Pr*(*V*_0_|*Class*_*k*_) and *Pr*(¬*V*_0_|*Class*_*k*_), where ¬*V*_0_ means “any value different than *V*_0_”

These subtle changes to the general process generates what we call the Refined Model, that is the final result of training and is returned as the solution to the steganalysis problem at hand.

#### Step 5: Model validation

This step simply classifies a set of objects that was reserved from the original labeled data, and reports the results in terms of classification success rate. This is the expected success rate for the classifier on new data that might be presented in the future.

## Experimentation

In this section we apply our framework, as described in the previous section, to datasets created with 11 different steganography schemes. We then use the results generated to evaluate the success of our approach. For eight of these schemes we obtained results that we seem either important or promising, while for the remaining three our approach was not successful. We discuss the results on these latter approaches as future work.

First we describe the data generation process. Data generation is critical for creating a robust experimental setting, because an incorrect generation process would lead to the detection of phantom vulnerabilities, i.e. introduced by the datasets (such as the detection of repeated passwords or predictable file names, etc.), instead of real vulnerabilities of the steganography schemes themselves. Then, we describe how we decide the values of some parameters that are of importance in our framework. After that, we present the global results over all datasets, followed by a detailed description of the actual solutions found, and how to use them to automate detection for each steganography scheme. A detailed false positive analysis is presented in S2 Appendix.

### Data generation

Traditional models in steganalysis often rely on knowing everything about the system with the exception of a secret key that is shared between the two parties, this is known as Kerckhoff’s Principle [24]. Our steganalytic approach, instead, can be considered as a blind attack with a known cover [25]. The framework does not assume any pre-existing knowledge on how each stego-system fundamentally operates. We train a classifier that works by extracting features from both cover and stego-objects to build a distinguisher but does not rely in any preliminary knowledge or assumption on the steganographic algorithms used.

This approach allows to apply the same procedure to unknown or new stego-systems, because no particular prior knowledge of the features of the systems is needed to built the models.

To ensure that our process of dataset generation did not introduce any bias into the solution, we kept passwords and embedded contents unique to each object. For each embedding scheme, we generated 100 pairs of stego-objects and cover-objects from our own repositories (such as the Kent Digital Media Archive), and from archive.org and other sources. The passwords were created randomly using the service at http://randomkeygen.com, using different password tables as sources (256-bit WEP, 156-bit WEP, 128-bit WEP, 64-bit WEP, ‘Decent Passwords’, etc.).

Contents to be embedded were created randomly thanks to the service at https://www.random.org/bytes/. Content size varied between 1-byte and 10KB. Our process thus ensures that each message and key was unique inside a given dataset. Table 7 lists the actual tools used for embedding.

**Table 7:** Steganography tools used in the experiments.

Name	Version	URL
F5		https://github.com/matthewgao/F5-steganography
OpenPuff	4.0	http://embeddedsw.net/OpenPuff_Steganography_Home.html
OurSecret	2.5.5	Only available from 3^rd^ parties
OmniHide Pro	1.0	http://omnihide.com/
Masker	7.5	http://www.softpuls.com/masker/
DeepSound	2.0	http://jpinsoft.net/deepsound/
Pixelknot	1.0.0	https://guardianproject.info/apps/pixelknot/

It can be argued against the generality of the use of only 100 pairs of objects. In this sense we have been limited in the size of the datasets for practical reasons, as the whole process of collecting, encoding data, etc. had to be repeated for each of the analyzed tools. Let’s suppose that we identify a given sequence of bytes as consistently found in all stego-objects for a given tool. Later, when testing on a different collection of objects, that sequence may not match any object if it included some bytes that are linked to a specific encoding option that was not used in our collection. If our system detected additional sequences for this tool this may not affect the result, but in a general case this is an obvious problem. There is no easy way to go around this problem besides training the model with larger and more diverse collections of objects, which is why we present our results as a methodology or framework. In this sense, in this work we report results that may be general but are open to improvement.

### Experimental methodology

For each dataset, we performed the experimental procedure in Algorithm 1. We used N-fold cross validation. This method splits the available data in *N* disjoint subsets called folds, and then performs *N* experiments. On each experiment, one fold is reserved to evaluate the model (Validation Set) and the rest is used for training. The procedure generates *N* models as a solution. Results for the *N* experiments are averaged to provide a final overall result for the procedure. For all our experiments we have used *N* = 10.

As a measure of accuracy we are using the classification success rate, that is, the average number for the sum of true positives (stego objects correctly classified) and true negatives (cover objects correctly classified). This measure is calculated by testing the trained model on a validation set that was randomly extracted from our data set and was not used for neither attribute extraction nor model generation.

**Algorithm 1**. Experimental procedure for N-fold cross-validation experiments with variable constant length of patterns (*N* = 10 for our experiments)

Sample data into *N* arrays *TRN*, *TST*, *VAL*

**for** *fold* = 1 to *N* **do**

 *cnstlen* ← 10

 **while** *success* < *Threshold* **do**

  Patts ← ∅

  **for** *cover*, *stego* ∈ TRN **do**

   Patts ← Patts ∪ *extract*(*cover*, *stego*, *cnstlen*)

  **end for**

  Patts ← Patts ∪ *generalize*(Pats)

  Model ← ∅

  **for** *pattern* ∈ Patts **do**

   **for** *cover*, *stego* ∈ TRN **do**

    Model ← Model ∪ *getCPT*_0_(*pattern*, *cover*)

    Model ← Model ∪ *getCPT*_1_(*pattern*, *stego*)

   **end for**

  **end for**

  Model ← *refine*(Model)

  *success* ← *test*(Model, TST)

  *cnstlen* ← *cnstlen* − 1

 **end while**

 Models[*fold*] ← Model

 Results[*fold*] ← *test*(Model, VAL)

**end for**

*result* ← *average*(Results)

**return** *result*, Models

This procedure calls several functions:

- *extract* is the function that searches for patterns.
- *generalize* is the function that creates the generalized patterns.
- *getCPT*_*i*_ is the function that calculates the conditional probability tables for each class *i*.
- *refine* performs Model Refining.
- *test* classifies a set of labeled objects and obtains a result in terms of success rate.

The Pattern Extraction phase requires two parameters: the length of the pattern constant part (*cnstlen*), both for Flag Replacement patterns and for EOF code injection patterns. For Flag Replacement another parameter is required, *varlen*, the maximum length of the variable part. The actual length of the variable part (*varlen*) was allowed to take values between 1 and 5 for all the experiments.

The value of *cnstlen* is important, because longer patterns are less likely to produce false positives. However, searching only for long patterns reduces the chance of finding consistent signatures. In order to set the best value for this parameter, we started with a maximum value and generated a candidate model. Then, this model was tested on a separate set of objects (Test Set) that were not used for training. If the success rate obtained on this Test set did not reach a certain threshold, we refine the training process decreasing the value of *cnstlen* in fixed steps. Otherwise, the train process stops and the model is considered final.

Also, in the Pattern Extraction phase, the search can explore all or only a part of each file. Preliminary experimentation found that it was enough to search 1 KB of data at the start of the file (or at the start of the injected code, generally towards the end of the file) to attain good results. Thus, we explored these two sections for each of the files in the pair. This limit is optional, and can be increased depending on the available computational resources. Also if specific knowledge of file structure for a format is available, search can be directed to the specific sections of the file that are known to be likely to be modified as a side result of the embedding process (headers, etc.). For the subsequent phases, Model Generation and Validation, the whole file was always considered: that is, we search for these patterns on the whole length of the file.

For the clustering mechanism used for pattern generalization, a distance threshold (*Th*) has to be defined. This is the maximum percentage of bytes that may differ in patterns that belong to the same cluster (intra-cluster distance). We empirically set a value of *Th* = 0.20 after some experimentation, as it tended to lead to good results.

Table 8 summarizes the parameters used in the experiments.

**Table 8:** Parameters used in our experimental procedure.

*cnstlen*	*varlen*	Explored	*Th*
10 → 2 (steps of -1)	{1, 2, 3, 4, 5}	1 KB	0.20

### Experimental results

We will show in this Section our new findings, corresponding to three different steganographic tools that had no previous known attacks. We will also include our results over a number of other tools with published weaknesses, and in these cases we provide due citation.

Our experiments were performed using 10-fold cross-validation. Results are the average success rate for the 10 folds. In Table 9 we provide the success rate attained in the experiments we consider successful, and for which we performed further analysis in section “Discussion on the solutions”. In these cases we obtained a success rate of practically 100% for most of our data sets, which means that we can automatically find vulnerabilities related to weak coding of the steganographic tools.

**Table 9:** Average success rate for successful experiments.

Tool	Method	Format	Success Rate	Refs.
Verification of previous results				
F5	F5 Scheme	JPEG	100%	[26]
OpenPuff	Flag Replac.	MP4	100%	[7]
OurSecret	EOF Data Inj.	MP4	100%	[8]
OmniHide Pro	EOF Data Inj.	MP4	100%	[8]
Masker	EOF Data Inj.	AVI	100%	[8]
—New results—				
OpenPuff	Flag Replac.	FLV	100%	
DeepSound	Unknown	WAV	99.5%	
Pixelknot	F5 Scheme	JPEG	100%	

Conversely Table 10 shows that our method was not able to obtain satisfactory results in all the analyzed steganography schemes, due to several reasons, for the OpenPuff tool. Partial results are commented in depth in “Future work on the schemes that we could not break”.

**Table 10:** Average success rate for unsuccessful experiments.

Tool	Format	Matched files	Success Rate	Comments
OpenPuff	MPEG	80% in extended dataset	99.5%	False negatives Base data set not general
OpenPuff	MP3	47.5% of our data set	100%	Results not general enough
OpenPuff	WAV	0%	No patterns found	

Even when this automatic process can be useful as an attack, true insight on the nature of the vulnerability requires in our opinion further explanation of the results. Sometimes we obtain models capable of detection of stego-objects, sometimes the classifier is identifying a regularity in the cover-objects. In some cases a single regularity is found that is able to perform classification, while in others several different regularities were found and had to be combined in order to detect the use of steganography. Therefore, in order to be certain that the resulting solutions are truly useful, the results have to be analyzed case by case. Thus, we are including a detailed description of a representative solution for each of the analyzed steganography schemes in the following section.

### Discussion on the solutions

This section shows and discusses the relevant parts of the models we were able to find for the data sets for which we obtained satisfactory results. We are not listing all the patterns in those models, as the training process generates many redundant patterns. Instead, we provide the “best” patterns in each model. This section is included in order to provide useful sequences for detection of stego-objects that allows in some cases to avoid the use of a full Bayes classifier, and instead requires only a simple “signature detection” mechanism.

Best patterns are selected manually using one of the following criteria: either they match the greatest number of files in the corresponding data set (that is, the *generality* of the pattern), or they have a high success rate over the files they actually match. In Tables 11–18 we provide two values: “matches” means the percentage of objects of the proper type where the pattern was found, while “success” is the classification success rate measured only on those matched objects.

**Table 11:** Best patterns found for the OpenPuff FLV tool.

Pattern		Matches	Distribution	Success
1	0000 0012 0001 3600	79.0%	Cover:	100%
	0000 ???? ???? 0200		0000 0000	
	0a6f 6e4d 6574 6144		Stego: ¬ 0000 0000	
2	0141 0900 0039 0000	68.0%	Cover:	100%
	0000 ???? ???? 0000		0000 0017	
	0000 014d 001f ffe1		Stego: ¬ 0000 0017	
Both patterns 1 or 2		100.0%		100%

**Table 12:** Best patterns found for the OpenPuff MP4 tool.

Pattern	Matches	Distribution	Success
6600 0000 1c64 7265	100%	Cover:	100%
6600 ???? ???? 0000		0000 0000	
0100 0000 0c75 726c		Stego: ¬ 0000 0000	
6961 0000 0020	100%	Cover:	99%
6d64 6864		0000 0000	
???? ????		Stego: ¬ 0000 0000	

**Table 13:** Best patterns found for the DeepSound WAV tool. Probability values are listed behind each of the alternative values for a feature.

Pattern	Matches	Values	Prob.	Success
1000 6461 7461 bc** **** ???? ???? ???? ???? ???? ???? ????	100%	Cover:		100%
		0000 0000 0000 0000 0000 0000 0000	0.96	
		feff feff 0200 0200 0400 0400 0000	0.02	
		0000 0000 0400 0040 0030 0030 0200	0.02	
		Stego:		
		0400 0400 0500 0300 0400 0300 0400	0.98	
		f4ff f4ff 0500 0300 0400 0300 0400	0.02	

**Table 14:** Best patterns found for the F5 JPEG tool.

Pattern	Matches	Values	Success
ffd8 ???? ???? ???? ???? ???? ???? ????	100%	Cover: ffe1 ¬ 0010 4a46 4946 0001 0000 0001 Stego: ffe0 0010 4a46 4946 0001 0000 0001	100%

**Table 15:** Best patterns found for the Pixelknot JPEG tool.

Pattern	Matches	Values	Success
ffd8 ???? ???? ???? ???? ???? ???? ????	100%	Cover: ffe1 ¬ 0010 4a46 4946 0001 0100 0001 Stego: ffe0 0010 4a46 4946 0001 0100 0001	100%

**Table 16:** Best patterns found for OurSecret MP4 tool.

	Matches	
Pattern	Cover	Stego
99b8 c178 720f 88dd dc34 2b4e 7d31 7fb5 e870 39a8	0%	100%
c178 720f 88dd dc34 2b4e 7d31 7fb5 e870 39a8 b842	0%	100%
720f 88dd dc34 2b4e 7d31 7fb5 e870 39a8 b842 7568	0%	100%
Plus the rest of the patterns included in the 40-octet signature		

**Table 17:** Best patterns found for OmniHide Pro MP4 tool.

	Matches	
Pattern	Cover	Stego
2020 2020 2020 2020 2020 2020 2020 2020 2020 2020	0%	100%

**Table 18:** Best patterns found for Masker AVI tool.

	Matches	
Pattern	Cover	Stego
9a9e 8ddb 5073 c2bf 65b8 1e03 aed5 62c6 cc71 9ab9	0%	100%
8ddb 5073 c2bf 65b8 1e03 aed5 62c6 cc71 9ab9 b948	0%	100%
5073 c2bf 65b8 1e03 aed5 62c6 cc71 9ab9 b948 49d2	0%	100%
Plus the rest of the patterns included in the 72-byte signature		

#### OpenPuff FLV

Though we were able to detect many patterns that were useful to classify some of the files, no pattern alone matched all files and provided a 100% success rate. In Table 11 we show that patterns 1 and 2 are some of the patterns that were found more commonly. These two patterns achieve a 100% success rate if combined in a single model such as our Naive Bayes model. That is why we report a results of 100% for this tool in Table 9.

Pattern 1 is related with the default null values located in the FLV header, to describe streamID. Research into FLV steganography through metadata tags has been discussed before in the academic literature, showing that stego-systems can embed data into metadata tags without corrupting the file whilst maintaining a good degree of imperceptibility [27, 28].

A method that ensures detection of stego-objects over the FLV scheme would use both patterns, 1 and 2, as in the following:

- If the sequence 0000 0012 0001 3600 0000 ???? ???? 0200 0a6f 6e4d 6574 6144 is found and any value in the indicated positions is **not** 0000 0000; *OR*
- If the sequence 0141 0900 0039 0000 0000 ???? ???? 0000 0000 014d 001f ffe1 is found and the value of those positions is **not** 0000 0017, then the object can be classified as a stego-object.

This method covers 100% of the files and achieves a 100% success rate in distinguishing cover from stego objects with this tool. Thus, it can be considered a new steganalytic result over this tool and format.

#### OpenPuff MP4

In this case we were able to find a single pattern that matched all files and provided 100% success. We also found some auxiliary patterns that were also very successful. These results are shown in Table 12.

These results are consistent with the findings published in [7] for the OpenPuff MP4 steganography scheme.

Using the patterns that has a 100% success rate would yield the following rule for quick classification: All stego-objects include the pattern 6600 0000 1c64 7265 6600 ???? ???? 0000 0100 0000 0c75 726c where the value of the variable part is not 0000 0000.

#### DeepSound WAV

The tool DeepSound (of Mr. Robot TV Series fame) proved to be more of a challenge. For DeepSound, patterns extracted straight away from a pair of cover and stego-objects were never found in any different pairs. When pairs of stego and cover objects were analyzed, we found patterns that were never consistent over more than one pair.

However, in this case good results were obtained by introducing the generalization process, as all the patterns that were found in separated pairs of objects could be automatically combined in the extended pattern shown in Table 13. Bytes that had different values across our data in that pattern were replaced by the general sign “*” and are not used to extract the attribute value. This model was able to achieve perfect accuracy results in our experiments.

There were, however, two cover-objects and one stego-object whose values for this feature were different from the rest. This posed a problem for the cross-validation experiments, that did not reach 100% success for the folds where those files were not used for training, thus the resulting value of 99.5% in Table 9. This problem was the result of the limited number of objects used for training (see section “Data generation”). The reason for reporting 99.5% accuracy was thus related to fact that we had a small number of objects of these types. It may be related to characteristics of the cover object, but further analysis is pending to provide insight of the singularity of these objects.

Table 13 describes one of the models that used those objects for training, where the aforementioned problematic values were included.

From these results we can define a simple method for the detection of steganography with DeepSound. In every stego-object the pattern 1000 6461 7461 bc** **** **** **** 0500 0300 0400 0300 0400 can be found (the values of the ‘*’ positions should not be taken into account). Note that because we have two different attribute values for the stego-objects, for this detector we have also replaced part of the value with the wildcard character (*). This may be useful to avoid any bias that we might have introduced in our data generation procedure. This pattern matched all the available stego-objects and none of the cover-objects, and is thus far the first steganalytic result reported on DeepSound.

#### F5 JPEG

For this tool, we found a pattern that matches every file pair and achieves a 100% success rate in classification. This pattern is shown in Table 14. Cover-files have random values after the first byte of the file, while stego files have a constant string as shown in the Table.

The signature found by our system was ffd8 ffe0 0010 4a46 4946 0001 0000 0001, which is the start of a constant block of data inserted by the F5 algorithm implementation. This block then contains a known and relatively long signature that translates to the ASCII string “JPEG Encoder Copyright 1998, James R. Weeks and BioElectroMech.” and was previously reported in [26]. If the extraction tool is run with the aim of searching for patterns with a longer variable part, it manages to extract the complete long signature, further showing the viability of our approach. This signature was not found in cover objects, where the block starts with the value ffd8 ffe1 and then continues with different, unrelated values.

We must note that this result indicates a problem with our data generation process. The header ffd8 ffe1 corresponds to cover-objects in EXIF format, but the tool generates JFIF format, where that header changes to ffd8 ffe0. Because we didn’t include files of JFIF format in our cover-files, our method includes the value ffd8 ffe1 as constant part of this signature. If our data had included cover-files of both types (EXIF and JFIF) files for output, the model would have included two patterns, each one matching one of the two versions of the header.

#### Pixelknot JPEG

Pixelknot is an popular steganography application for Android devices, available at the Google Play store, which is part of *The Guardian Project*, a group of developers that make open-source secure mobile apps for everyone but particularly tailored for Journalists, Human Rights Activists and citizens in repressive regimes. Their web is at https://guardianproject.info. This issue has been responsibly disclosed to their team before publication.

For Pixelknot, which had no previous reported weaknesses, we were able to detect the exact same vulnerability than for F5. The signature found can also be used to classify files with a 100% success rate (see Table 15). This is not entirely surprising, as the developers recognise their tool is based on F5. What is interesting is that the long pattern present in F5 is not present in Pixelknot, but despite this slight improvement in security, other identical patterns are present and found by our framework.

#### OurSecret MP4, Omnihide MP4 and Masker AVI

We have applied our approach to three algorithms known to use EOF Data Injection. We generated models that were able to achieve 100% accuracy in each case.

Our framework was able to extract several constant patterns for these files. In all cases patterns matched all the stego-files and never matched any of the cover-files. This is, of course, the best case scenario for the steganalyst.

In Tables 16–18, as we don’t have attributes, we just list the probability of matching cover-objects or stego-objects.

- For OurSecret, we were able to find a signature that had previously been found (manually) by previous work on this steganographic scheme [8]. In Table 16 we list the first 10-byte patterns detected, which can be joined to form the full signature 9e97 ba2a 0080 88c9 a370 975b a2e4 99b8 c178 720f 88dd dc34 2b4e 7d31 7fb5 e870 39a8 b842 7568 7191.
- For OmniHide, we detected a sequence of bytes with value 2020 in stego-objects that was not present in any cover-object. Although this sequence can be used to classify our data with 100% success rate, as a signature it is problematic as it is relatively low entropy, so has the potential to lead to many false positives. It simply encodes a series of blank spaces to accommodate file names, whose hexadecimal code is 0x20. We carried out a test for false positives to determine the chance of this signature appearing naturally in MP4 files. These results can be seen in S2 Appendix.
- For Masker, we found the signature 3030202020202020, that was consistently present in the initial 1*KB* of the stego-object. This pattern can be used for classification, though it can also be seen as a weak detector due to its low entropy. It can be expected that it might lead to an large percentage of false positives. So we continued trying to find different signatures, and succeeded in finding a longer signature that appears in a variable position in each of the stego-objects. This finding is consistent with previous results in [7]. The full 73-octet string that can be used as signature is:
  9e8d db50 73c2 bf65 b81e 03ae d562 c6cc 719a b9b9 4849 d264 ee49 b763 7bef eba1 0104 407b f0ed 2d86 47e1 5ffc 7c41 c98b c902 ca1c 0721 ee8d 3266 477c 8fe1 4ee8 ae66 ab32 e3d2 f9a1 0e.
  In Table 18 we show the three first patterns found that are part of that signature.

### General discussion on the merits and limitations of our approach

We have proposed an automatic approach for system steganalysis that compares favorably to the manual approaches used so far in the literature. This is a novel way of performing steganalysis that has the additional advantage of being valid across different tools and formats, and is powerful enough to lead to new steganalytic results or, at times, replicate existing ones. These techniques assume the presence of implementation vulnerabilities within the embedding components of a steganography tool. It is, therefore, surprising how effective they are.

Experimental results shown that our approach to steganalysis was able to detect the presence of different types of signature across several different steganographic schemes, without any prior assumptions of which kind of scheme was used. Although our pattern extraction mechanism could be extended using a more powerful grammar for pattern definition, the current version was enough to provide good results over several tools. However, not all the tools have this kind of vulnerability, and therefore these methods are complementary of the statistical attacks developed for each media type. Moreover, findings by our method are specific of a given tool, and may change as the tool is updated over time.

In our experimental setting, there were some practical limitations due to the computational resources available. For instance, if modifications are introduced into unexamined object areas, we would not be able to locate them. We generally had to limit our search to small sections both at the beginning and the end of the files. This practical limitation can be overcome by either increasing computational effort dedicated to pattern extraction, or by implementing a more efficient search strategy. We are currently exploring both paths.

Another practical limitation is due to the fact that our framework operates at the byte-level. Certain software might only introduce bit-level changes that might not be easily recognized by our software.

Though the Naive Bayes Classifier has performed well in our setting, it has obvious limitations from a theoretical perspective if features do not show the property of conditional independence. However our domain knowledge suggests that, because the changes are largely unintentional, this assumption works for at least some of the features detected by our framework. This limitation can be overcome by learning these dependencies from the training data and constructing a more complex Bayes Network when generating the model.

We have detected that Data Generation is a tricky matter. If a vulnerability is not traced back to the tool code (which is not generally available), it may happen that the vulnerability found is linked to certain characteristics of the cover file. Universality of the results, that is, whether the findings are applicable to every object generated using a given tool could be improved by increasing the number and diversity of files in the data set. This would provide our method with representative training examples for a wider range of possibilities. However even in the case that generated data does not cover all cases (for instance, when a given signature is not present in 100% of the examples of a given tool), partial success is useful to trace the vulnerability in code.

## Conclusion and future work

This research emphasizes a key point in the field of steganography: Steganalysis should be extended beyond the scope of the embedding algorithm, to study the impact of steganography on the whole stego-object. Any file imprint, be that from the scheme itself or from an implementation component can compromise the security of the core steganography scheme. We have observed empirically that many libraries for image processing leave traces of their use in the resulting images. This is, in many cases, due to old copyright messages that are no longer relevant but can still severely impair the security of the stego system, as seen with F5.

General improvements to the embedding operation are a counter to both statistical and system attacks. It is interesting to note that patterns or signatures found with our model are generally easier to remove when the algorithm developer wants to patch their system, compared with statistical attacks that may expose issues that can not be addressed without devising an entirely new scheme.

Our method can be used to perform a blind classifier for any kind of media format, that detects the use of steganography, exploiting the fact that the embedding tools may either introduce regularities in the stego-object, or destroy regularities in the cover-object. Both types of regularities may be used for detection. The proposed approach can work reliably even with a limited number of training cases, and it is flexible enough so that it can scale with the computational resources available, in order to increase accuracy as needed.

The vulnerabilities automatically found by our framework were consistent with the results already published for part of the analyzed schemes, but we were also able to carry out successful steganalysis against schemes for which no vulnerabilities had been previously discovered.

However, our results also show that special care has to be exerted in the application of the method in order to use enough data for training, in order to have representatives of all variations on the attacked media format. This was a problem detected when reporting the signatures for some of the schemes, because parts of those signatures would only be found in a subset of all the possible files for a given media format.

Even when our framework was unsuccessful in its steganalytic analysis of certain tools, it was still shown to be useful from a reverse engineering perspective. In cases where it could not consistently identify signatures, the framework was capable of identifying the exact parts of a file that were modified by the stego-system. This provides us with a fundamental understanding of how the stego-system under investigation operates. It can be a good starting point in a forensic investigation, as it provides valuable early insights that may lead to more significant findings.

As noted in the detailed discussion on each scheme, our framework is able to obtain many patterns that can be joined in a successful model for classification, not just isolated signatures. However we have not implemented a specific way of selecting the best patterns in the model. This step is not straightforward, as every pattern can be characterized by a set of possibly conflicting properties: for instance, we have mentioned the number of matches and the success rate in matched files. Other features may be of relevance for further applications: patterns with low entropy will be more prone to false positives; long patterns (that is, patterns with long constant parts) generally are less subject to false positives but have lower generalization abilities. These aspects can be evaluated using a multi-criteria approach that can be used for model refining or even during the model construction step, to ensure that the resulting classifier uses the best possible combination of patterns. This will be explored in future works.

### Future work on the schemes that we could not break

There were some steganographic schemes where our results were not satisfactory. However, we were able to find some regularities that can be exploited in order to improve the proposed framework.

- For OpenPuff MPEG, we were able to find patterns capable of classifying all files with great accuracy, achieving a success rate of 99.5%. This is due to the model generated in one of the folds being unable to classify just one of the test files. This file was correctly classified with the rest of the models generated in cross-validation.
  However, these results did not generalize well. The patterns detected depend, as we concluded after further analysis, on particular details of the compression mechanism of the MPEG file. When tested on additional objects taken from a different source, 20% of those objects were not matched by the model. For that reason, we consider that our work on this stenographic scheme is not concluded yet.
  In Table 19 we show a model with a single pattern that is able to classify the remaining 80% of the provided objects correctly. Considering only these “vulnerable” objects, cover-objects always matched the pattern and had one of three different specific values that were never present in the stego-objects. Note that 25% of the “vulnerable” stego-objects were not matched by the pattern (we return the value *False* in this case), but this situation was never found in cover-objects.
  These results can be jointly used to design a method to detect files with hidden info embedded with this scheme. Note that in this case we are not providing simply a signature that matches any stego-object. Instead the method should search the file for the string 01ba 4400 0400 0401 43** ???? 0000 01bb 000c **** **** ???? c0c0 20e0 e0e6 0000 01c0 (where the * can be any value). If the value extracted for this pattern is either 2bf8 21ff, 3bf8 21ff or ebf8 21ff, the mpeg corresponds to a cover-object. If the value is different, or the pattern does not match the object, it is a stego-object produced with OpenPuff.
- For OpenPuff MP3 files, we also found patterns that provided 100% accuracy but did not match all the available objects. The detected regularity was only found in 47% of the files after searching the first 4096 bytes of the files. This result is not good enough for us, because we require perfect (or near perfect) accuracy, as discussed in the Introduction.
  For roughly half of the object pairs in the dataset, we detected a constant string that was consistently found in cover-objects but was never found in stego-objects. This string (0000 3483 8000 004c 414d 4533 2e39) can be used to definitely classify an object as cover-object, if present.
  An in-depth analysis of the objects matched by this pattern showed that the steganography scheme under study systematically inserted a block of data inside the stego-object. We detected that, for the cases where this signature is found, the cover-object had a section of data that started with 414d 4533 2e39 3155 5555 or 414d 4533 2e39 3255 5555 and was filled up to the length of 256 bytes with a series of 5555 constant byte values. The steganographic process removed the filling bytes and inserted a block of 256 bytes just after the four initial bytes (0000 3483 8000 004c), ending it with the rest of the signature (414d 4533 2e39) which was shifted exactly 256 bytes away from its original position.
  Even if our framework was unable to break this steganography scheme, it provided us with useful insights as to how this flawed stego-system fundamentally operates.
- For OpenPuff WAV, the pattern extraction mechanism was unable to find any consistent patterns. Thus, it was unable to successfully perform classification. This may mean that either our definition of pattern was insufficient powerful to describe the kind of modifications performed by the steganography scheme, or that this scheme has no vulnerabilities that can be detected by a system attack, thus being a good candidate for a targeted statistical attack.

**Table 19:** Best patterns found for the OpenPuff MPEG tool. Frequencies listed calculated over the 80% of the objects that were vulnerable to the attack.

Pattern	Matches	Distribution	Prob.	Success
01ba 4400 0400	100%	Cover:		100%
0401 43** ????		2bf8 21ff	0.33	
0000 01bb 000c		3bf8 21ff	0.18	
**** **** ????		ebf8 21ff	0.49	
c0c0 20e0 e0e6	75%	Stego:		
0000 01c0		None of the above	0.75	
		False	0.25	

## Supporting information

S1 Appendix Appendix I. Example of Classification using a Naive Bayes Model.
(PDF)

S2 Appendix Appendix II. Experimental Validation.
(PDF)
