# Correction: System steganalysis with automatic fingerprint extraction

**DOI:** 10.1371/journal.pone.0200457

**Published:** 2018-07-05

**Authors:** The PLOS ONE Staff

The following information is missing from the Funding section: This work was supported by European Union’s Horizon 2020 Research and Innovation Programme, Grant agreement No.700326. The publisher apologizes for the error.
