# Supplementary material for: System steganalysis with automatic fingerprint extraction
Source: PLoS One. 2018 Apr 25;13(4):e0195737. doi: 10.1371/journal.pone.0195737 (PMC5919007; doi:10.1371/journal.pone.0195737)
Supplement: S1 Appendix — Example of Classification using a Naive Bayes Model. (PDF) [file pone.0195737.s001.pdf]

System Steganalysis with Automatic Fingerprint Extraction

Alejandro Cervantes[1*], Tom Sloan[2], Julio Hernandez-Castro[2], Pedro Isasi[1]

1 University Carlos III of Madrid, Leganés, Madrid, Spain
2 University of Kent, Canterbury, United Kingdom

* Corresponding author: Email: acervant@inf.uc3m.es

Appendix I: Example of classification using a Naive Bayes model

Let us assume we are testing a case with the example model in section 'Step 3: Model generation". For each pattern in the model we obtain two factors. One is the (estimated) likelihood for cover-objects, and the other for the stego-objects. These products of likelihoods are used as in Eq. 4 to obtain the class.

Using this example, we would first test Pattern 1. If the pattern does not match the file, we consider the value as "False". If it matches, then we inspect its value for that case, and go to the proper part of the Model in the example to find the factors to be used in Eq. 4:

$$Class(X_i) = \arg \max_{k \in \{0,1\}} \prod_{p \in Model} Pr(Class_k) \times Pr_p(A_{ip}|Class_k) \quad (4)$$

- If the value is `0000 0000`, we use the following values:

$$\begin{aligned} Pr(\texttt{0000 0000}|Class_0) &= 0.9 \\ Pr(\texttt{0000 0000}|Class_1) &= 0.18 \end{aligned}$$

- If the value is different than `0000 0000` we use:

$$\begin{aligned} Pr(\neg(\texttt{0000 0000})|Class_0) &= 0.1 \\ Pr(\neg(\texttt{0000 0000})|Class_1) &= 1 - 0.18 = 0.82 \end{aligned}$$

  , where $\neg$(`0000 0000`) means that the value is **different** from `0000 0000`.

Then we proceed to pattern 2, where we determine whether the pattern matches the file ($True$) or not ($False$).

- If the pattern matches the file, we use $Pr(True|Class_0) = 0.0$ and $Pr(True|Class_1) = 1.0$. Usual practice in these classifiers is to *smooth* values that are equal to 0, because any factor whose value is 0.0 would make the rest of the patterns useless for classification. The simplest method found in literature is to replace 0.0 for a small, non-zero value [14].

- However, if the pattern does not match the file, we use $Pr(False|Class_0) = 1.0$ and $Pr(False|Class_1) = 0.25$.

So let's say that a file has `0000 0000` for the first pattern and the second pattern **matched** this file; then we would calculate:

- For $Class_0$,

$$\begin{aligned}
Pr(& \texttt{0000 0000}|Class_0) = \\
= & \; Pr_1(\texttt{0000 0000}|Class_0) \times Pr_2(True|Class_0) = \\
= & \; 0.9 \times 0.0 = 0.0 \\
Pr(& Class_0|\texttt{0000 0000}) = \\
= & \; \alpha \times Pr(Class_0) \times Pr(\texttt{0000 0000}|Class_0) \\
= & \; \alpha \times 0.5 \times Pr(\texttt{0000 0000}|Class_0) \\
= & \; \alpha \times 0.5 \times 0.0 = 0
\end{aligned}$$

- For $Class_1$,

$$\begin{aligned}
Pr(& \texttt{0000 0000}|Class_1) = \\
= & \; Pr_1(\texttt{0000 0000}|Class_1) \times Pr_2(True|Class_1) = \\
= & \; 0.18 \times 0.75 = 0.135 \\
Pr(& Class_1|\texttt{0000 0000}) = \\
= & \; \alpha \times Pr(Class_1) \times Pr(\texttt{0000 0000}|Class_1) \\
= & \; \alpha \times 0.5 \times Pr(\texttt{0000 0000}|Class_1) \\
= & \; \alpha \times 0.5 \times 0.135 > 0
\end{aligned}$$

Because we are training with balanced classes, the values of $P(Class_0)$ and $P(Class_1)$ are both 0.5 and the factor α is the same for both. Thus, instead of calculating the value for the posterior probability ($P(Class_0|\texttt{0000 0000})$) we can simply compare the likelihoods (0 and 0.135) and conclude that the file should be classified as $Class_1$, the class that has the greater likelihood.
