# Supplementary material for: System steganalysis with automatic fingerprint extraction
Source: PLoS One. 2018 Apr 25;13(4):e0195737. doi: 10.1371/journal.pone.0195737 (PMC5919007; doi:10.1371/journal.pone.0195737)
Supplement: S2 Appendix — Experimental Validation. (PDF) [file pone.0195737.s002.pdf]

System Steganalysis with Automatic Fingerprint Extraction

Alejandro Cervantes[1*], Tom Sloan[2], Julio Hernandez-Castro[2], Pedro Isasi[1]

1 University Carlos III of Madrid, Leganés, Madrid, Spain
2 University of Kent, Canterbury, United Kingdom

* Corresponding author: Email: acervant@inf.uc3m.es

Appendix II: Experimental validation

A last component of our analysis should be a study of how many false positives the proposed signatures will lead to.

We can approximate the probability of appearance of a single random byte string S (the signature) of length "l" inside a longer random byte string F (the file) of length "L". The simplest approximation to this probability can be calculated using Eq.5.

$$P(FP) = 1 - P(\neg \mathrm{S} \in \mathrm{F}) \approx 1 - (1 - 2^{-8\times l})^{L} \quad (5)$$

For files of $L = 1$ GB this probability drops below $1E - 06$ for signatures as short as $l = 7$ bytes in length (our signatures are always longer). However, this approach can underestimate the probability of random appearance of some strings, because the assumptions of randomness in signatures and files are both incorrect. In case of files with high compression rates,we might assume randomness, but that can be misleading because not every segment of the file is compressed. As a trivial example, the probability of appearance of specific strings (e.g., '0000') in a generic file format is far higher from the the theoretical. That is the reason why additional empirical validation has been conducted.

For each format and tool we have obtained a signature for, we examined a large number of files obtained from various sources in which we did not expect any steganographic contents. This is to determine whether the proposed signatures are likely to occur naturally inside cover files. Table 20 shows the signatures used in these tests.

Table 20. Test signatures for the false positive analysis

Tool	Number of Tests	Signature
F5 JPEG	2000	ffd8 ffe0 0010 4a46
		4946 0001 0000 0001
OpenPuff FLV	1000	$\neq$ 0000 0000 and 0000 0017
OurSecret MP4	2000	See Table XIII
OmniHide Pro MP4	2000	2020 2020 2020 2020 2020
Masker AVI	1000	3030 2020 2020 2020
OpenPuff MP4	2000	Atom detection
OpenPuff MPEG	1000	Arbitrary values
Pixelknot JPEG	2000	ffe0 0010 4a46 4946
		4946 0001 0100 0001
DeepSound WAV	1000	0500 0300 0400 0300 0400

For each test a new batch of cover files were used. JPEG images were downloaded from the Dresden Image Database [1]. Videos were tested across MP4, AVI, FLV and

MPEG formats. These files were obtained from YouTube (25% of files), Archive.org (50% of files) through an automatically generated query, and our own KDMA archive (25% of files). Audio WAV files were obtained from WAVsource.com.

Table 21 shows the results of these tests.

Table 21. Tests for false positives across known signatures

Tool	Method	Format	False Positive Rate
F5	F5 Scheme	JPEG	0%
OpenPuff	Flag Replac.	MP4	0%
OurSecret	EOF Data Inj.	MP4	0%
OmniHide Pro	EOF Data Inj.	MP4	0%
Masker	EOF Data Inj.	AVI	0%
OpenPuff	Metadata	FLV	0.4%
OpenPuff	Metadata	MPEG	0%
Pixelknot	F5 Scheme	JPEG	0%
DeepSound	Unknown	WAV	0%

The false positives for Masker only consider the shorter signature. If we use the full 73-byte signature there is a rate of zero false positives. For OpenPuff FLV, the 0.4% rate for false positives comes from corrupted FLV files downloaded from Archive.org.

References

1. Thomas Gloe and Rainer Böhme, The 'Dresden Image Database' for Benchmarking Digital Image Forensics., In: Proceedings of the 25th Symposium On Applied Computing (ACM SAC 2010); 2010. 2. p. 1585–1591

10
